# What is the potential for bisexual men in China to act as a bridge of HIV transmission to the female population? Behavioural evidence from a systematic review and meta-analysis

**DOI:** 10.1186/1471-2334-11-242

**Published:** 2011-09-15

**Authors:** Eric PF Chow, David P Wilson, Lei Zhang

**Affiliations:** 1 The Kirby Institute for infection and immunity in society, University of New South Wales, Sydney, Australia; 2 CFI Building, Corner of West and Boundary Streets, Darlinghurst, Sydney NSW 2010, Australia

**Keywords:** Men who have sex with men (MSM), meta-analysis, bisexual behaviour, condom use

## Abstract

**Background:**

HIV prevalence among men who have sex with men (MSM) in China has rapidly increased in recent years. It is suggested that MSM could be a potential bridge of HIV transmission to the general female population. We investigated the bisexual behaviour of MSM in China through systematic review and meta-analysis.

**Methods:**

We conducted a systematic review and meta-analyses on published peer-reviewed Chinese and English literature during 2001-2010 according to the PRISMA guidelines. Marital status and sexual behavioural indicators of MSM were presented graphically using forest plots. The pooled effect rates with 95% confidence intervals were also calculated. Meta-regression analyses were performed to examine the factors associated with high heterogeneities across the studies.

**Results:**

Forty-three eligible articles (11 in English and 32 in Chinese) were identified. Our results showed that 17.0% (95% CI: 15.1-19.1%) of MSM in China are currently married to a woman and 26.3% (95% CI: 23.6-29.1%) of MSM had female sexual partners in the last six months. The pooled estimates for condom use rate between MSM and female sex partners was 41.4% (95% CI: 35.5-47.5%) at the last sex act; and 25.6% (95% CI: 23.0-28.4%) in the last six months. The consistent condom use rates with regular, non-commercial, casual and commercial female sex partners in the last six months were 23.3% (95% CI: 11.25-42.1%), 39.0% (95% CI: 28.8-50.3%) and 55.8% (95% CI: 41.4-69.4%), respectively.

**Conclusions:**

A substantial proportion of Chinese MSM is currently married or had sexual relations with a female in the past six months. In addition, low condom usage was common between married MSM and their wives, hence posing a higher risk of transmitting HIV. Harm-reduction programs targeting married MSM and their female partners are necessary to curb the further spread of HIV infection to the general female population.

## Background

Although blood transfusions and injecting drug use had historically been the dominant risk factors associated with HIV infection in China, sexual transmission has become the main route of HIV transmission in recent years. Among the 48,000 (41,000-55,000) Chinese people newly diagnosed with HIV in 2009 [1], 42.2% and 32.5% were due to heterosexual and homosexual transmission respectively [1,2]. Notably, HIV prevalence among men who have sex with men (MSM) in China had increased rapidly from 1.4% to 5.3% between 2001-2009 [3] and this increasing trend is likely to continue in coming years.

The rapid increase in HIV prevalence among Chinese MSM was probably due to many factors including the increasing numbers of men who engage in homosexual sex; multiple male partners; and high levels of unprotected anal intercourse. A recent systematic review and meta-analysis study showed that just over one-third of MSM report consistent condom use with their male sex partners in the last six months and only 20% consistently used condoms with their regular male partners [4]. It has also been reported that sexually active Chinese MSM report having an average of six to seven male sexual partners in the last six months and 18% of them had participated in group sex in the past 12 months [5].

Due to traditional cultural and family values, Chinese MSM often marry to conceal their homosexuality from family and friends. It was estimated that approximately 50-70% of MSM have had sex with females in their lifetime [6-10] and 70-90% will eventually marry [11,12]. This suggests that bisexual behaviours among Chinese MSM are common. As they are often the only son in their families, as a direct result of China's one-child policy, they also face their parents' expectation to have children to continue the family line.

Chinese MSM tend to have low rates of condom use with their female partners [13]. Given the rapid increase in HIV prevalence among Chinese MSM, their bisexual behaviours could potentially act as a bridge of HIV transmission to the general population [5,9,14-16]. In this study we conducted a systematic review of studies on bisexual behaviours among Chinese MSM in both Chinese and English literature to examine the extent to which bisexual behaviour is common among Chinese MSM.

Our review, coupled with meta-analyses for specific questions, summarised evidence of bisexual behaviours of Chinese MSM. These included the age of MSM who are currently married; the percentage of Chinese MSM who had female sexual partners in last six months; the percentage of MSM who are currently married; and the condom usage rate among MSM with their female sexual partners at the last sex act or in the last six months. Different types of partnerships, including regular, non-commercial casual or commercial, were also distinguished in our review and analysis. In comparison with a recent published systematic review on behavioural studies among Chinese MSM [17], this study employed a meta-analysis approach with a strong focus on the condom use with different types of female partners. In addition, this study substantially extended the previous analysis to include both English and Chinese literature. Our study provides more detail insights and quantifiable results on the bisexual behaviours among MSM in China.

## Method

### Searching strategy

Two investigators (EPFC, LZ) performed a systematic literature search for peer-reviewed studies from the following electronic databases: PubMed, Wanfang Data, China National Knowledge Infrastructure (CNKI) and Chinese Scientific Journals Fulltext Database (CQVIP), published between 2001 and 2010. The keywords searched in the databases were ("unprotected sex" *OR *"condom use" *OR *"risk behaviour") *OR *("KABP [knowledge, attitudes, beliefs and practices]" *OR *"behaviour") *OR *("Married" OR "Unmarried") *OR *("female sexual partners") *AND *("homosexual" *OR *"MSM" *OR *"men who have sex with men" *OR *"gay") *AND *"China". The references in the relevant articles were searched manually. We limited our search to articles published in Chinese and English only. We followed the PRISMA (Preferred Reporting Items for Systematic Reviews and Meta-Analyses) guidelines issued in 2009 for conducting and reporting this systematic review and meta-analysis [18].

### Study selection

Studies were included if they (1) reported the percentage of condom use (at the last sex act and/or consistent condom use in the last six months) *OR *(2) reported the percentage of MSM who had female sexual partners in last six months, through a peer-reviewed publication of cohort or cross-sectional study. We excluded studies in which the target study population was specifically money boys or MSM drug users only. Review papers, non-peer reviewed theses, local reports, conference abstract and presentations were excluded from this study. The two duration types of condom use in "last sex act" and "in last six months" were the most frequent and standardised reported indicators in most Chinese and English literature. Therefore consistent condom use rates within other durations (e.g. past one, three, twelve months or others) were also excluded. Studies which did not report study location, time periods and sample size were also excluded. If the study was duplicated in the databases, studies published in Chinese or published latest were excluded from this review.

### Quality assessment

The quality of studies was assessed using a validated quality assessment tool for cross-sectional studies [19]. The following seven items were assessed to calculate a total quality score: (1) clear definition of the target population; (2) representativity of probability sampling; (3) sample characteristics matching the overall population; (4) adequate response rate; (5) standardised data collection methods; (6) reliable and valid survey measures/instruments; (7) appropriate statistical methods. Answers were scored 0 and 1 for 'No' and 'Yes', respectively. The total quality score varied between 0 and 7 (see Table 1).

**Table 1:** A summary of studies reporting bisexual behaviour among men who have sex with men in China

	Study Design						Sexual orientation % (n/N)		Condom usage % (n/N)			Quality Score
				
First author, published year	Study Period (MM/YY)	Study location	Age range (Mean)	Recruitment method^1^	**Sampling method **^2^	Sampling size	Percentage of married MSM	Had female partners in last six months	Types of female partner^3^	Condom use at last sex act	100% condom use in last six months	
**East China**												

Cai GF, 2008 [26]	06/2006-12/2006	Zhejiang	16-46	MSM Venues	-	152	16.4% (25/152)	18.9% (28/148)	Commercial	-	33.3% (2/6)	4

Chen SC, 2007 [25]	07/2005-09/2005	Hangzhou	18-70 (28.0)	MSM Venues, internet invitation	Snowball	365	26.8% (97/362)	11.5% (42/365)	Commercial	75.0% (3/4)	44.4% (4/9)	4

Choi KH, 2007 [39]	09/2004-06/2005	Shanghai	18-56 (28.0)	MSM Venues	Snowball	477	13.0% (62/477)	25.2% (120/477)	-	-	-	6

Fu LJ, 2007 [23]	02/2006-12/2006	Zhejiang	19-41 (26.0)	-	Snowball	55	18.2% (10/55)	16.4% (9/55)	-	-	-	3

Hu Q, 2006 [6]	08/2005	Jiangxi	15-60 (25.0)	MSM Venues, internet advertisement	Snowball	200	10.5% (21/200)	-	Overall	51.1% (48/94)	-	4

Liao MZ, 2006 [27]	09/2005-11/2005	Shandong (Jinan, Yantai, Weihai)	18-73 (26.0)	MSM Venues	-	157	12.1% (19/157)	22.9% (36/157)	Commercial	78.6% (11/14)	50.0% (7/14)	3

Ruan S, 2009 [16]	03/2007-07/2007	Jinan	N/A	Peer referral	RDS	428	16.1% (69/428)	*Total: *33.6% (144/428) [*Married: *97.1% (67/69); *Unmarried: *21.4% (77/359)]	-	-	-	5
	
	04/2008-06/2008	Jinan	N/A	Peer referral	RDS	500	25.4% (127/500)	*Total: *32.6% (163/500) [*Married: *78.0% (99/127); *Unmarried: *17.2% (64/373)]	-	-	-	

Wu J, 2008 [52]	10/2006-12/2006	Shanghai	18-54 (26.6)	MSM Venues	-	203	11.8% (24/203)	43.3% (88/203)	Overall	47.7% (42/88)	34.1% (30/88)	4

Yang HT, 2006 [8]	05/2004-07/2004	Jiangsu	18-56 (28.0)	MSM Venues, internet advertisement	-	222	21.6% (48/222)	45.9% (102/222)	Overall	-	21.3% (23/108)	5

Zhu YW, 2007 [53]	03/2006-07/2006	Jinan	17-66 (25.4)	CDC	-	400	11.0% (44/400)	20.0% (80/400)	Overall	-	27.5% (22/80)	4

**Northeast China**												

Gu Y, 2004 [54]	03/2003-04/2003	Shenyang	16-49	MSM Venues	-	342	-	36.3% (124/342)	-	-	-	3

Liu LY, 2008 [55]	2006	Mudanjiang	N/A (29.0)	MSM Venues	-	202	16.8% (34/202)	-	Overall	26.3% (20/76)	24.0% (12/50)	2

Wang J, 2008 [56]	08/2006-10/2006	Harbin	N/A	MSM Venues	Convenience	401	-	22.4% (90/401)	Overall	48.9% (44/90)	-	3

Wang L, 2006 [57]	07/2004	Heilongjiang	17-66	MSM Venues	-	397	12.6% (50/397)	24.7% (98/397)	-	-	-	3

Zhang D, 2007 [58]	2002	Harbin	18-67	MSM Venues	-	215	-	41.9% (90/215)	-	-	-	5
	
	2004	Harbin	18-67	MSM Venues	-	397	-	24.7% (98/397)	-	-	-	
	
	2006	Harbin	18-67	MSM Venues	-	647	-	25.0% (162/648)	-	-	-	

Zhao H, 2009 [59]	01/2008-10/2008	Harbin	17-52 (25.0)	VCT Clinic	-	89	7.9% (7/89)	14.6% (13/89)	Overall	53.8% (7/13)	30.8% (4/13)	2

**North China**												

Guo W, 2008 [60]	10/2007-11/2007	Hebei	18-54	MSM Venues, internet chat rooms	-	118	17.9% (19/106)	33.0% (35/106)	-	-	-	3

Liu H, 2001 [61]	08/2000-10/2000	Beijing	16/72 (30.1)	MSM Venues	TLS	84	29.8% (25/84)	27.4% (23/84)	-	-	-	3

Liu H, 2005 [10]	09/2001-01/2002	Beijing	18-69 (27.0)	MSM Venues	-	481	9.4% (45/481)	30.1% (145/481)	Overall	-	27.6% (40/145)	4

Liu H, 2007 [28]	08/2005-12/2008	Beijing	18-55 (27.2)	MSM Venues	-	416	11.5% (48/416)	31.7% (132/416)	Commercial	-	75.0% (12/16)	4
								
									Regular	-	16.5% (16/97)	
								
									Non-commercial casual	-	40.7% (22/54)	

Ma J, 2007 [62]	04/2006-10/2006	Tianjin	N/A (25.2)	Internet advertisement	-	433	9.5% (41/433)	22.9% (99/433)	-	-	-	5

Qu L, 2009 [63]	10/2008-12/2008	Inner Mongolia	18-63 (27.0)	MSM Venues	-	604	21.4% (129/604)	19.7% (119/604)	Overall	28.6% (34/119)	19.3% (23/119)	2

Ruan Y, 2007 [30]	06/2005-11/2005	Beijing	17-54 (26.2)	MSM Venues, peer referring, internet advertising	Snowball, convenience	526	6.5% (34/526)	10.6% (56/526)	Regular	-	32.6% (14/43)	5

									Non-commercial casual	-	34.8% (8/23)	

Wang CH, 2007 [12]	06/2003-10/2006	Chengde	15-28	MSM Venues	Snowball	82	26.8% (22/82)	22.0% (18/82)	-	-	-	1

Wang XL, 2009 [64]	11/2008-12/2008	Tangshan	19-53	MSM Venues	-	114	50.0% (57/114)	50.9% (58/114) -		-	-	2

**South Central China**												

Cai YM, 2009 [65]	10/2007-12/2007	Shenzhen	18-45 (27.0)	MSM Venues	RDS	351	21.9% (77/351)	28.5% (100/351)	Overall	-	36.8% (35/95)	3

He Q, 2005 [66]	04/2003-05/2003	Guangzhou	18-55	Peer referral, internet advertisement	Snowball	121	-	21.5% (26/121)	Overall	-	26.9% (7/26)	4

Li N, 2007 [40]	2006	Henan	17-68 (28.3)	CDC	Continuous	187	31.6% (59/187)	36.9% (69/187)	-	-	-	2

Liu H, 2009 [45]	2007	Shenzhen	18-45	MSM Venues	RDS	293	24.9% (73/293)	25.9% (76/293)	Overall	-	31.1% (91/293)	5

Xing JM, 2007 [41]	10/2005-12/2005	Hunan	14-65 (29.8)	MSM Venues, internet invitation	-	372	27.3% (101/370)	29.0% (108/372)	Overall	-	32.7% (33/101)	3

Xu YF, 2008 [67]	08/2007-09/2007	Nanning	17-38	Internet advertisement, peer referral	-	230	6.1% (14/230)	13.9% (32/230)	-	-	-	2

Zhong F, 2009 [64]	05/2008-08/2008	Guangzhou	≥ 18	VCT Clinic	RDS	379	21.9% (83/379)	28.2% (107/379)	-	-	-	5

**Northwest China**												

Miao ZF, 2009 [68]	04/2008-06/2008	Yinchuan	18-55 (27.7)	MSM Venues, internet	Snowball	312	24.4% (76/312)	37.5% (117/312)	Overall	47.0% (55/117)	-	2

Zhang M, 2009 [24]	05/2008-06/2008	Ürumqi	18-54 (27.4)	-	RDS	231	11.3% (26/231)	16.9% (39/231)	-	-	-	1

**Southwest China**												

Feng L, 2009 [13]	07/2006-11/2006	Chongqing	≥ 18	MSM venues, community outreach, peer recruitment, web-based recruitment	VBS, CABC	1000	18.2% (182/1000)	24.5% (245/1000)	Overall	36.3% (89/245)	23.7% (58/245)	5
	
	07/2007-11/2007	Chongqing	≥ 18	MSM venues, community outreach, peer recruitment, web-based recruitment	VBS, CABC	1044	22.7% (237/1044)	25.2% (263/1044)	Overall	28.5% (75/263)	22.4% (59/263)	

Feng Y, 2010 [69]	03/2007-07/2007	Chengdu	16-44	MSM Venues	Snowball	538	19.2% (96/500)	26.5% (136/513)	-	-	-	6

Lau JT, 2009 [70]	2005	Kunming	15-75	MSM Venues, peer referral, internet recruitment	VBS, snowball	387	20.4% (79/387)	36.4% (141/387)	-	-	-	5
	
	2006	Kunming	15-75	MSM Venues, peer referral, internet recruitment	VBS, snowball	316	11.4% (36/316)	30.1% (95/316)	-	-	-	

Wang Y, 2008 [29]	12/2006-01/2007	Mianyang	16-57 (24.8)	MSM Community	RDS	201	7.5% (15/199)	12.9% (26/201)	Overall	57.7% (15/26)	42.3% (11/26)	4

									Commercial	87.5% (7/8)	62.5% (5/8)	

Xiao Y, 2009 [71]	07/2006-09/2007	Chongqing	18-68 (27.7)	MSM Venues, internet recruitment, community outreach, peer referral	VBS, snowball	1692	17.1% (289/1692)	21.6% (364/1689)	Overall	35.8% (130/363)	23.5% (85/362)	6

Zhou J, 2008 [72]	09/2006-12/2006	Guiyang	15-49 (24.0)	Peer referral, internet recruitment	-	406	5.2% (21/406)	7.4% (30/406)	Overall	63.3% (19/30)	-	5

**Others**												

Xiao Y, 2010 [73]	07/2006-09/2006	20 cities from 7 provinces (Gansu, Inner Mongolia, Heilongjiang, Jilin, Liaoning, Ningxia, Chongqing)	15-68 (28.4)	Internet advertisement, community outreach, peer referral, MSM Venues	Snowball, VBS	4983	27.2% (1354/4983)	26.0% (1298/4983)	Overall	-	18.2% (231/1266)	6

Zhang BC, 2007 [74]	2004	6 cities (Chongqing, Shenyang, Dalian, Qingdao, Nanjing, Xi'an)	15-72 (27.6)	MSM Venues	Snowball	1389	17.9% (249/1389)	42.4% (589/1389)	Overall	-	23.6% (139/589)	5

Zhang BC, 2008 [5]	2005-2006	9 cities (Harbin, Shenyang, Xi'an, Zhengzhou, Shanghai, Nanjing, Chongqing, Wuhan, Chengdu)	13-78 (29.1)	MSM Venues	Snowball	2250	24.7% (555/2246)	*Total: *52.1% (548/1051) [*Married: *74.0% (344/465); *Unmarried: *34.8% (204/586)]	Overall	-	*Total: *22.6% (190/839) [*Married: *16.2% (65/401); *Unmarried: *28.5% (125/438)]	5

^1 ^MSM Venues: including gay bars, parks, sauna, bathroom, dance clubs; VCT Clinic: HIV voluntary counseling and testing clinic

^2 ^RDS: Respondent-Driven Sampling; TLS: time-location sampling; VBS: Venue-based sampling, CABC: Cruising area-based convenience sampling

^3 ^Overall partnerships represent the combination of regular, non-commercial casual and commercial female partnerships.

### Data abstraction

For all identified studies, we also extracted information about the study: first author and year of publication, study period, study location, age of MSM, study base, sampling method, sample size, marital status (percentage of currently married MSM), and types of female sexual partners (regular, non-commercial casual, commercial or overall). We divided the studies into six traditional administrative regions (East China, Northeast China, North China, South Central China, Northwest China and Southwest China). See Table 1 for a description of included studies and extracted data.

### Quantitative data synthesis

The data were analysed in the Comprehensive Meta-Analysis software (V2.0, Biostat, Englewood, New Jersey). For every stratified analysis, we tested the significance of heterogeneity by the Cochran's *Q *statistics (*p *< 0.10 indicates significant heterogeneity) and the level of heterogeneity was assessed by the *I*^2 ^statistic [20]. The value of the *I*^2 ^statistic indicates low (25%), moderate (50%) and high (75%) heterogeneity between studies. Due to the presence of heterogeneity in different stratified analyses, random effect models were used to compute the pooled effect rates (i.e. percentage of married MSM, percentage of MSM who had female sexual partners in the last six months and the condom use rates), 95% confidence intervals (CI) and relative weights of studies. Results were graphically presented in forest plots. Publication bias was tested by the Begg and Mazumdar rank correlation (*p *< 0.05 on the Kendall's tau indicates significant publication bias) where three or more studies contributed to a stratified analysis [21].

Heterogeneity in meta-analysis is a test of the variation between studies. A high level of heterogeneity (*I*^2 ^≥ 75.00, *p <*0.10) may be due to the factors of the size of study, sampling methods and study base [20]. We investigated factors related to heterogeneity among studies by using meta-regression [22]. Due to insufficient number of studies, we could only perform meta-regression for the meta-analysis on: (1) MSM marriage rate; (2) MSM who had female sexual partners in the last six months; (3) condom use between MSM and female partners at last act; and (4) consistent condom use between MSM and female partners in the last six months. Potential study characteristics associated with high heterogeneity were examined in a multiple variables model. The multiple variables used in this study were the language of publication (Chinese versus English), size of study (< 200 versus ≥ 200), recruitment method (MSM venues versus non-MSM venues versus not reported), sampling method (not reported method versus other methods versus RDS (respondent driven sampling) or snowball method), and study time period (early 2000s (2000-2004) versus late 2000s (2005-2009)). The regression coefficient and *p *values (*p *value < 0.10 indicates that the factor is significantly associated with heterogeneity) for each study characteristic on meta-analysis were calculated by the STATA statistical software package (version 10). Results of meta-regression and subgroup meta-analyses based on the potential study characteristics were summarised in Table 2.

**Table 2:** Result of individual variable meta-regression models for each stratified meta-analysis

	**Stratified meta-analyses**							
	
**Study Characteristic**	**Marital Status of MSM**		**Had female partners** **in last six months**		**Condom use** **at last sex act**		**Consistent condom use** **in last six months**	
			
	**Pool estimate %** **(95% CI), n**	**Meta-regression** **(β, *p*-value)**	**Pool estimate %** **(95% CI), n**	**Meta-regression** **(β, *p*-value)**	**Pool estimate %** **(95% CI), n**	**Meta-regression** **(β, *p*-value)**	**Pool estimate %** **(95% CI), n**	**Meta-regression** **(β, *p*-value)**
			
Language of article:								
Chinese	16.4 (13.9-19.3), n = 29	0.187	25.8 (21.7-30.4), n = 30	0.058	45.4 (37.5-53.6), n = 9	**-0.282**	26.9 (23.8-30.2), n = 13	0.009
English	18.2 (15.2-21.5), n = 13	*p *= 0.312	26.8 (24.2-29.5), n = 16	*p *= 0.686	33.6 (29.0-38.6), n = 3	***p *= 0.058**	23.4 (19.1-28.4), n = 5	*p *= 0.960
			
Sample size:								
< 200	19.8 (13.1-28.9), n = 10	**-0.335**	25.9 (19.8-33.2), n = 10	-0.056	45.4 (37.5-53.6), n = 9		28.6 (24.7-32.8), n = 11	-0.313
≥ 200	16.3 (14.3-18.4), n = 32	***p *= 0.090**	26.3 (23.4-29.5), n = 36	*p *= 0.730	33.6 (29.0-38.6), n = 3	-	23.2 (20.4-26.3), n = 7	*p *= 0.139
			
Recruitment method:								
MSM venues	18.7 (16.6-21.0), n = 30		28.6 (25.5-32.0), n = 33		38.4 (32.8-44.3), n = 9		25.1 (22.5-27.9), n = 15	
Non MSM venues	12.6 (8.6-18.1), n = 10	**-0.203**	21.1 (16.2-26.9), n = 11	**-0.139**	59.4 (47.4-70.3), n = 3	**0.368**	34.2 (23.6-46.6), n = 3	0.107
Not reported	13.5 (8.4-21.0), n = 2	***p *= 0.035**	16.8 (12.9-21.6), n = 2	***p *= 0.059**	-	***p *= 0.068**	-	*p *= 0.679
			
Sampling method:								
RDS/snowball	17.2 (14.5-20.2), n = 17		25.7 (21.7-30.3), n = 21		45.9 (36.4-55.7), n = 4		25.8 (21.8-30.4), n = 8	
Other methods	21.7 (18.1-25.8), n = 8	0.013	26.5 (22.9-30.3), n = 5	0.009	37.0 (27.4-47.7), n = 3	0.122	27.5 (19.7-36.9), n = 2	0.089
Not reported	14.6 (11.1-19.0), n = 17	*p *= 0.886	26.6 (22.5-31.1), n = 20	*p *= 0.899	41.6 (28.6-55.9), n = 5	*p *= 0.128	24.9 (21.8-28.4), n = 8	*p *= 0.268
			
Time period:								
2000-2004	16.8 (12.2-22.9), n = 5	0.023	32.6 (27.0-38.7), n = 9	-0.236	-	-	24.1 (21.4-27.1), n = 4	0.093
2005-2009	17.1 (15.0-19.3), n = 37	*p *= 0.922	24.9 (22.1-27.9), n = 37	*p *= 0.134	41.4 (35.5-47.5), n = 12		26.1 (22.8-29.7), n = 14	*p *= 0.535

Table showing the pool estimate (%), 95% confidence interval (CI), number of studies (n), meta-regression coefficient (β) and significance of β (*p*-value). *p*-values in bold print represent significant associations (*p *< 0.10).

We also performed further analyses on the correlation between age and percentage of married MSM by the Spearman correlation coefficient (*r*).

## Results

### Trial Flow/Flow of included studies

**Figure 1:**
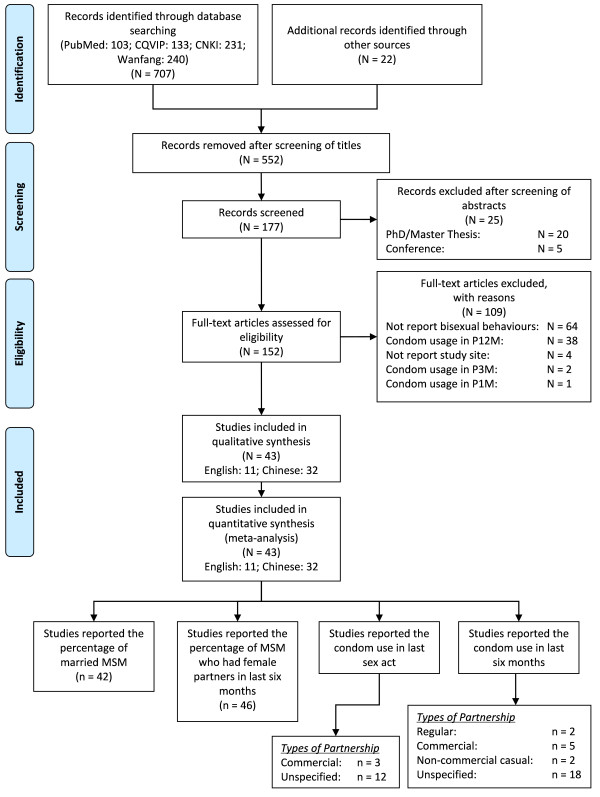
**Flow chart for selection of studies with number of articles (N) and number of estimates (n)**.

We identified 707 articles from four electronic databases (103 in PubMed, 133 in CQVIP, 231 in CNKI and 240 in Wanfang); we also identified 22 articles from an internet search and reference lists of published articles. Due to duplication of articles and unrelated topics, we excluded 552 articles after screening the titles. We screened the abstracts of the remaining 177 articles, following which 25 articles were excluded because 20 were non peer-reviewed theses and five were conference presentations or abstracts. The remaining 152 articles were eligible for full-text screening; we further excluded 109 articles (64 did not report bisexual behaviour; 38 reported condom use with female partners in last twelve months; four did not report the study site; two reported condom use in last three months and one reported condom use in the last one month only). Finally, we included 43 articles (11 in English and 32 in Chinese) in a qualitative and quantitative synthesis (Figure 1).

### Study characteristics

The sample size of identified studies ranged from 55 to 4983 (median: 365; IQR: 201-480). Among 43 studies, two did not identify the study base [23,24] and approximately 73% (30/41) of studies recruited MSM participants from MSM venues. Five studies [25-29] reported the condom use rate between MSM and female sex workers. Two studies [28,30] reported the condom use rate between MSM and regular/non-commercial casual female sexual partners. Only two studies [5,16] reported the percentage of married and unmarried MSM who had sex with female in the last six months. The majority of studies recruited MSM participants from MSM venues such as gay bars, sauna and bathrooms (Table 1).

### Quantitative data synthesis

#### Marital status

**Figure 2:**
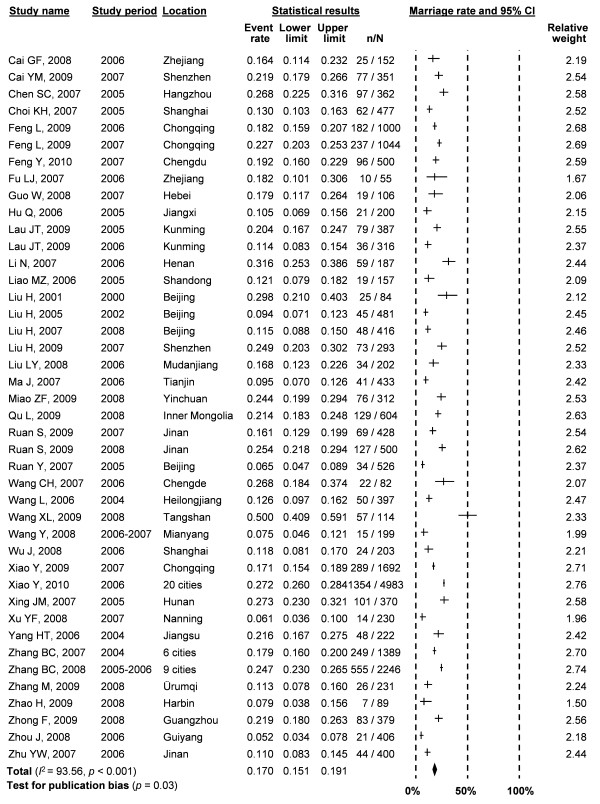
**Forest plot of pooled estimates of percentage of married MSM in China**.

**Figure 3:**
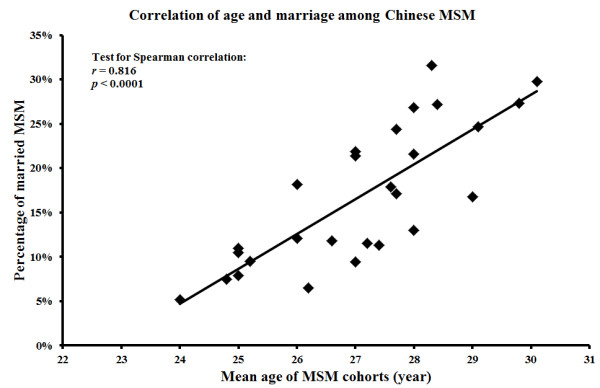
**Correlation between mean age of MSM in China and the percentage of MSM who are currently married**.

It was estimated that 17.0% (95% CI: 15.1-19.1%) of MSM in China are currently married (Figure 2). High and significant heterogeneity (*I*^2 ^= 93.56, *p *< 0.001) existed and the publication bias was marginally significant (*p *= 0.03). Meta-regression analysis showed that heterogeneity was explained by the size of study (*β *= -0.335, *p *= 0.090) and recruitment method (*β *= -0.203, *p *= 0.035) (Table 2). Studies with a small sample size (< 200) and participants recruited from MSM venues were more likely to report a higher marriage rate than large studies conducted in non-MSM venues. In addition, a significant association (*r *= 0.816, *p *< 0.0001) between the mean age of the sampled MSM cohorts and the current marital status (Figure 3) was observed, which indicates that older MSM are more likely to be married compared to younger MSM.

#### Bisexual behaviour of MSM

**Figure 4:**
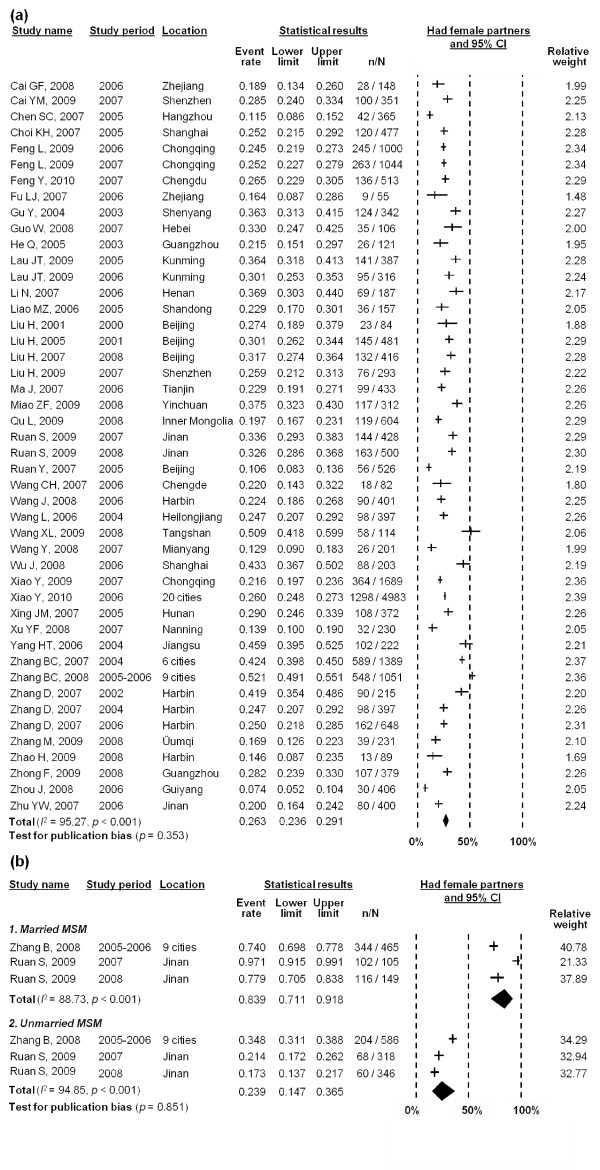
**Forest plot of pooled estimates of percentage of (a) MSM; and (b) married and unmarried MSM who had female partners in last six months**.

The random effects model revealed that approximately 26.3% (95% CI: 23.6-29.1%) of MSM had female sexual partners in the last six months (Figure 4a); no significant publication bias existed (*p *= 0.353). High and significant heterogeneity between studies existed (*I*^2 ^= 95.27, *p *< 0.001) in the meta-analysis. Only one study factor (recruitment method) was significantly associated with this heterogeneity (*β *= -0.139, *p *= 0.059) (Table 2). Studies which recruited participants from gay venues were more likely to have female sexual partners in the last six months than MSM recruited from non-gay venues.

A comparison of the bisexual behaviours between married and unmarried MSM showed that married MSM (83.9%, 95% CI: 71.1-91.8%) were more likely to have female sexual partners than unmarried MSM (23.9%, 95% CI: 14.7-36.5%) in last six months (Figure 4b) (χ^2 ^= 490.4, *p *< 0.001). No significant publication bias was observed (*p *= 0.851).

#### Condom use with female partners

**Figure 5:**
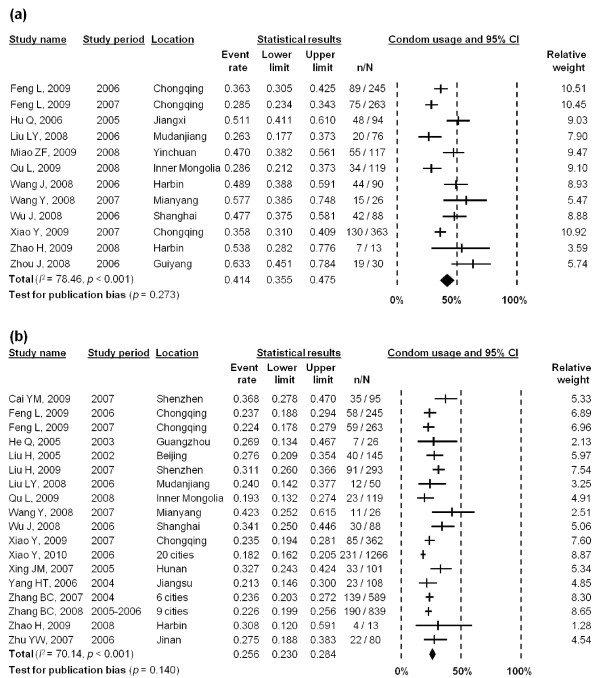
**Forest plot of (a) condom use with overall female partners in the last sex act; and (b) reported consistent condom use with overall female partners in last six months**.

The pooled estimate of levels of condom use between MSM and their female partners during 2005-2008 was 41.4% (95% CI: 35.5-47.5%) at last sex act (Figure 5a) with significant heterogeneity (*I*^2 ^= 78.46, *p *< 0.001) throughout these studies. Meta-regression analysis showed that two study characteristics, language of articles (*β *= -0.282, *p *= 0.058) and recruitment method (*β *= 0.368, *p *= 0.068), significantly contributed to this relatively high heterogeneity (Table 2). Since the sample size and time period were collinear with other study factors in the meta-regression model, these two study characteristics were both excluded by the meta-regression model. There was no significant publication bias in this meta-analysis (*p *= 0.273).

Consistent condom use with female sexual partners in last six months was 25.6% (95% CI: 23.0-28.4%) during the period of 2002-2008 (Figure 5b). Moderate heterogeneity was observed between studies (*I*^2 ^= 70.14, *p *< 0.001), but we found that none of the study characteristics was significantly associated with this heterogeneity (Table 2). There was no significant publication bias observed (*p *= 0.140).

**Figure 6:**
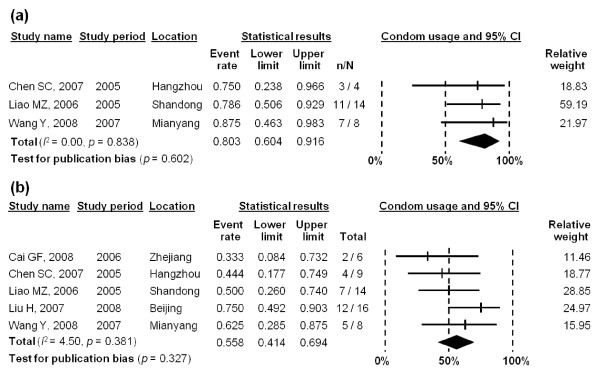
**Forest plot of (a) condom use with commercial female partners in the last sex act; and (b) reported consistent condom use with commercial female partners in last six months**.

Condom use among MSM with commercial female partners was also analysed according to identified studies (three reported condom use at last act [25,27,29], and five reported rates of consistent use in the last six months [25-29]). The pooled estimates of condom use in commercial partnerships were 80.3% (95% CI: 60.4-91.6%) (*I*^2 ^= 0.00, *p *= 0.602) at last sex act and 55.8% (95% CI: 41.4-69.4%) (*I*^2 ^= 4.50, *p *= 0.327) in the last six months (Figure 6). No significant publication bias was observed in either of the stratified analyses (*p *= 0.602 and 0.327 for last sex act and last six months, respectively). Since heterogeneities were low and not significant in both stratified meta-analyses, meta-regression analysis was not performed.

**Figure 7:**
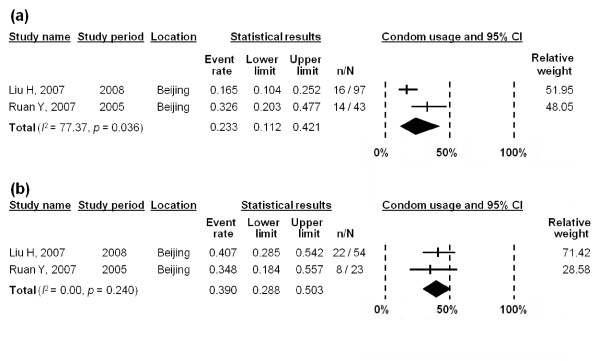
**Forest plot of condom use with (a) regular female partners; and (b) non-commercial casual female partners in last six months**.

Two studies reported the consistent condom use with regular and non-commercial causal female partners in the last six months [28,30]. In comparison with commercial partnerships, condom usage rates in regular and non-commercial casual partnerships were much lower, estimated to be 23.3% (95% CI: 11.25-42.1%) (*I*^2 ^= 77.37, *p *= 0.036) and 39.0% (95% CI: 28.8-50.3%) (*I*^2 ^= 0.00, *p *= 0.240), respectively (Figure 7). Publication bias could not be assessed due to lack of studies in both stratified meta-analyses.

## Discussion

Our meta-analysis showed that approximately 17.0% of MSM are currently married to a female in China, which is consistent with another recent review which reported an average of 17.5% [17]. In contrast, the percentage of MSM who are married in most Western developed countries is much lower. Only 1.5-3.0% of MSM are currently married to a woman in the United States [31-33] and 7.9% in Australia [34]; however, higher rates of marriage among MSM were observed in some other developing countries (for example, 35-42% in India [35,36]). There is generally greater acceptance and legitimacy of gay identity, gay marriage or cohabitation relationships and less social stigma, discrimination and family pressure in developed countries. Previous studies showed that only 11-17% of Chinese MSM disclosed their homosexual sexual orientation to their wives [37,38] and 1% of them disclosed to their children [37]. Due to the high prevalence of bisexual behaviours among MSM in China, this group is very likely to facilitate HIV transmission to the female population to a much greater extent than occurs in most countries where HIV predominantly affects MSM. Our review provides a timely synthesis of current evidence of bisexual behaviours among MSM in China, which could inform the implementation of effective intervention programs.

Previous available studies indicated that Chinese MSM had an average of 1-5 female sexual partners in the last six months [7,8,39,40]. Additionally, we estimated that a higher proportion (83.9%) of married MSM have had female sexual partners than unmarried MSM (23.9%). However, only 16.2% and 28.5% of married and unmarried MSM have had consistent condom use with their female partners in the past six months, respectively [5]. Fifty-eight percent of married MSM had concurrent male and female sexual partners in last six months, hence putting their wives at a greater risk of HIV infection [41].

Partnership type had strong influences on condom use of MSM. In general, condom usage at last sex act between MSM and overall female sexual partners was estimated to be 41.4% (95% CI: 35.5-47.5%); but only 25.6% (95% CI: 23.0-28.4%) of MSM used condoms consistently in the last six months. A study in 2004 found that 31.8% of MSM had regular female sex partners, 6.0% had non-commercial casual female sex partners and 4.5% had commercial female sex partners [14]. Among three specific partnership types, consistent condom use was estimated to be the greatest in commercial partnerships (55.8%, 95% CI: 41.4-69.4%), followed by non-commercial casual partnerships (39.0%, 95% CI: 28.8-50.3%) and regular partnerships (23.3%, 95% CI: 11.2-42.1%). In general, reasons of low condom use with partners mainly included: emotional and physical sensation, availability of condoms, and loyalty and trust [5,25]. In addition, sexual abuse was common in China. It was reported that 30% of wives were abused by their homosexual or bisexual husbands to relieve their mental pressure from social stigma and discrimination [42]. This indicated that regular sexual partners of Chinese MSM were often not empowered to negotiate for protected sexual intercourse with their male partners.

Several limitations of our study must be noted. First, the selected studies in this review recruited MSM participants predominantly from major urban Chinese cities. Bisexual behaviour such as MSM who have female sexual partners and condom usage may be significantly different between urban and rural regions. Second, based on our search criteria, we could not identify any studies on condom use at the last sex act in the South Central region or consistent condom use in the last six months in the Northwest region. Our pooled estimates of condom usage may not be representative for the entire MSM population in China. Third, very few studies reported bisexual behaviours stratified according to their marital status and partnership types. This substantially limited the strength of meta-analysis in these cases. Fourth, due to insufficient description of some studies, we were unable to classify whether the sexual contacts between MSM and female was penile-vaginal intercourse, penile-anal intercourse or oral sex. Male-to-female anal contacts often had a higher risk of HIV transmission than vaginal contact [43]. Due to reproductive reasons, MSM might not use condoms during vaginal sex but they may use condoms in anal sex. Since vaginal intercourse was more common than anal intercourse in male-female partnership, we assumed all condom usage estimates were associated with vaginal sexual intercourse. Fifth, due to limited number of studies available, we were unable to investigate the associations between the age of MSM and consistent condom use in different geographical locations and with different types of partnerships. For the same reason, the contribution of age to the heterogeneity of the studies could not be investigated. Sixth, our study only addressed the overall MSM population; specific populations such as male sex workers ('money boys') were not included in this study. In China, 'money boys' have become one of the emerging high-risk sub-populations in gay communities in recent years [44]. However, very few epidemiological and socio-behavioural studies have focused on this population to date. Limited studies indicated that Chinese 'money boys' are usually younger than the overall MSM population and unmarried [38,45-47]. An estimated 71% of Chinese money boys had 1-3 male clients and 11% had more than 10 clients per week [38]. Approximately 27% of money boys had female clients in last six months [48]. Unprotected sex with both male and female clients was very common (59.4% and 55.6%, respectively) [38]. A survey in 2007 found that 43% of the money boys had female sexual partners but only 36% reported consistent condom use in the last six months [45]. Money boys were more likely to move between major cities to earn extra income from the commercial sex industry and hence they may be a particularly important population for transmitting HIV in different geographical locations [49]. These characteristics may result in the population of money boys becoming the core group for HIV transmission among MSM and the general population [44,45]. Further investigations are necessary to understand the behaviours of this sub-population in China.

Moderate to high heterogeneities were observed in the conducted meta-analyses. Given the limited details of study information, we only investigated five potential study characteristics associated with the heterogeneity in the meta-regression model. Recruitment method was found to be the major study characteristic associated with high heterogeneity in the stratified meta-analyses (associated with marital status, percent of MSM who had female sex partners and condom use at last sex act). Furthermore, we postulate that several other factors, including geographical Chinese regions, age of MSM and migrant status, may also be associated with the heterogeneity observed, although these factors could not be assessed by meta-regression due to limited data. Other study variables such as the quality of interviewers, questionnaire or marital status of MSM were not reported in the collated studies but may also contribute to the heterogeneities across studies.

MSM is a population highly susceptible to the emerging HIV epidemic in China [9,50,51]. Further studies on specific sub-populations of MSM, such as money boys, transgender MSM, MSM who inject drugs and bisexual MSM, are necessary to understand their sexual behaviours and contribution to the HIV transmission within and beyond the MSM population. Currently, there are no specific public health interventions targeting bisexual MSM in China. Our results indicate that MSM recruited at MSM venues were more likely to be currently married, had sex with females in last six months, and had unprotected sex with females. Harm-reduction interventions such as distribution of condoms and lubricants to bisexual MSM may be implemented at MSM venues and further expanded to the wider communities. Given the low condom usage between MSM and their regular female partners, public health intervention strategies should particularly target married MSM and their regular female partners. Sexual behaviour and condom use rates with different partnerships should be clearly identified in the future social-behavioural surveys in order to understand the bisexua behaviour among MSM and to curb the spread of HIV transmission from MSM to the overall female population.

## Conclusion

Men who have sex with men are a population that is highly susceptible to the rapid spreading HIV infection in recent years. Our study shows that a substantial proportion of MSM is currently married or has had sex with a female in the past six months. Consistent condom use rates are low among partnerships with regular females. Our findings imply that the general female population, as regular partners of MSM, has a high risk of contracting HIV infection that are bridged from their bisexual male partners. Scale-up of the harm-reduction interventions and health educations for bisexual MSM and their female partners are necessary to halt the further spread of HIV into the general female population.

## Competing interests

The authors declare that they have no competing interests.

## Authors' contributions

All authors were involved in the study design, including setting up the keywords search and the project protocol. EPFC and LZ performed the literature search, quality assessment and data extraction. EPFC performed data analysis. LZ and DPW assisted with data analysis and interpretation. DPW was responsible for the supervision of the project. All authors contributed in writing and editing the manuscript. All authors read and approved the final version of the manuscript.

## Pre-publication history

The pre-publication history for this paper can be accessed here:

http://www.biomedcentral.com/1471-2334/11/242/prepub
